# Poly[(μ_6_-benzene-1,3,5-tri­carboxyl­ato-κ^6^ *O* ^1^:*O* ^1′^:*O* ^3^:*O* ^3′^:*O* ^5^:*O* ^5′^)tris­(*N*,*N*-di­methyl­formamide-κ*O*)tris­(μ_3_-formato-κ^3^ *O*:*O*:*O*′)trizinc(II)]

**DOI:** 10.1107/S1600536813028687

**Published:** 2013-10-26

**Authors:** Jaeung Sim, Taemin Kim, Jin Kuk Yang

**Affiliations:** a Department of Chemistry, Soongsil University, 369 Sangdo-Ro, Dongjak-Gu, Seoul 156-743, Republic of Korea

## Abstract

The asymmetric unit of the title compound, [Zn_3_(HCO_2_)_3_(C_9_H_3_O_6_)(C_3_H_7_NO)_3_]_*n*_, contains one Zn ion, one formate ligand, one *N*,*N*-di­methyl­formamide (DMF) ligand and one-third of a benzene-1,3,5-tri­carboxyl­ate (btc) ligand situated on a crystallographic 3 axis. Each Zn^II^ atom is coordinated by one O atom from a DMF ligand, two O atoms from two btc ligands and three O atoms from three formate ligands in a distorted octa­hedral geometry. The Zn^II^ atoms are connected by the formate and btc ligands, forming hexanuclear cores, which are linked by btc ligands, constructing a two-dimensional layered network along the *ab* plane.

## Related literature

For general background to compounds with metal-organic frameworks, see: Eddaoudi *et al.* (2000 ▶)·The crystal structures of isotypic compounds with Ni^II^ and Mg^II^ were reported by Maniam & Stock (2011 ▶) and Yeh *et al.* (2010 ▶), respectively.
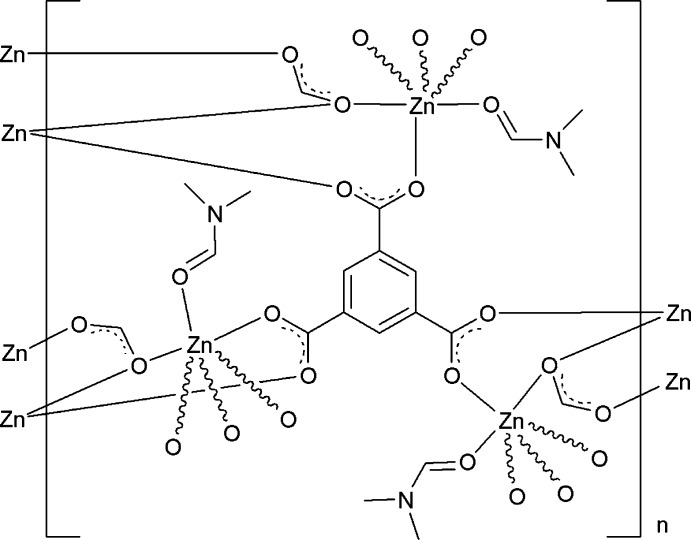


## Experimental

### 

#### Crystal data

[Zn_3_(HCO_2_)_3_(C_9_H_3_O_6_)(C_3_H_7_NO)_3_]
*M* *_r_* = 757.56
Trigonal, 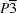

*a* = 13.8594 (17) Å
*c* = 8.0100 (14) Å
*V* = 1332.5 (4) Å^3^
*Z* = 2
Mo *K*α radiation
μ = 2.76 mm^−1^
*T* = 153 K
0.10 × 0.02 × 0.02 mm

#### Data collection

Bruker SMART APEX CCD diffractometer
Absorption correction: multi-scan (*SADABS*; Sheldrick, 2003 ▶) *T*_min_ = 0.770, *T*_max_ = 0.947
7964 measured reflections
2027 independent reflections
1563 reflections with *I* > 2σ(*I*)
*R*_int_ = 0.153

#### Refinement

*R*[*F* ^2^ > 2σ(*F* ^2^)] = 0.040
*wR*(*F* ^2^) = 0.089
*S* = 0.81
2027 reflections
127 parameters
H-atom parameters constrained
Δρ_max_ = 0.72 e Å^−3^
Δρ_min_ = −0.79 e Å^−3^

### 

Data collection: *SMART* (Bruker, 1997 ▶); cell refinement: *SAINT* (Bruker, 1997 ▶); data reduction: *SAINT*; program(s) used to solve structure: *SHELXS97* (Sheldrick, 2008 ▶); program(s) used to refine structure: *SHELXL97* (Sheldrick, 2008 ▶); molecular graphics: *CrystalMaker* (CrystalMaker, 2013 ▶); software used to prepare material for publication: *publCIF* (Westrip, 2010 ▶).

## Supplementary Material

Crystal structure: contains datablock(s) global, I. DOI: 10.1107/S1600536813028687/cv5429sup1.cif

Structure factors: contains datablock(s) I. DOI: 10.1107/S1600536813028687/cv5429Isup2.hkl

Additional supplementary materials:  crystallographic information; 3D view; checkCIF report

## Figures and Tables

**Fig. 1.**
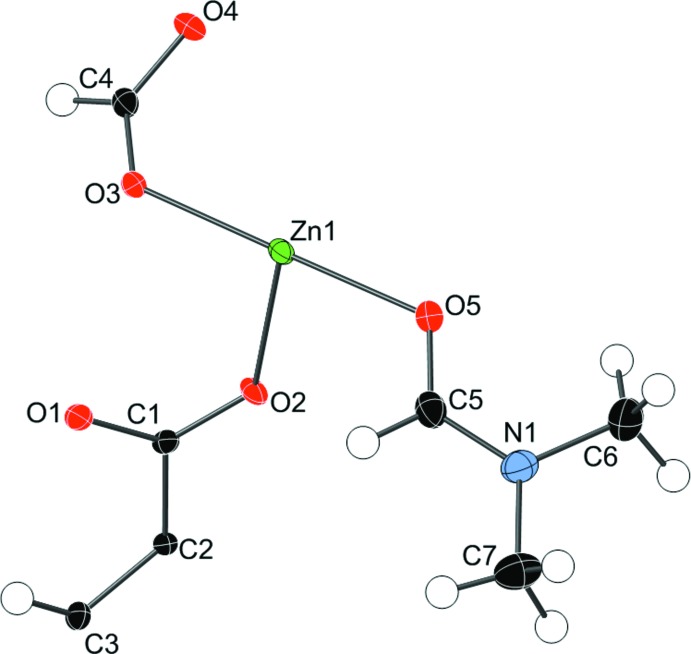
A content of the asymmetric unit of (I) showing the atomic numbering. Displacement ellipsoids are drawn at the 50% probability level. Hydrogen atoms are represented with empty balls without labels.

**Fig. 2.**
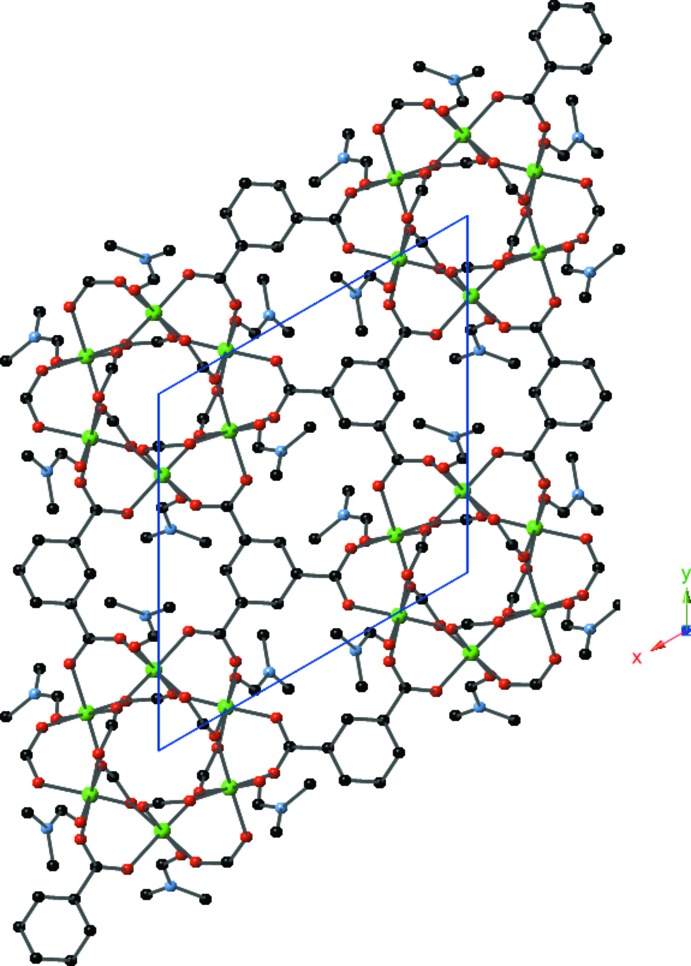
A portion of the packing diagram of (I) viewed along the c aixs.

**Fig. 3.**
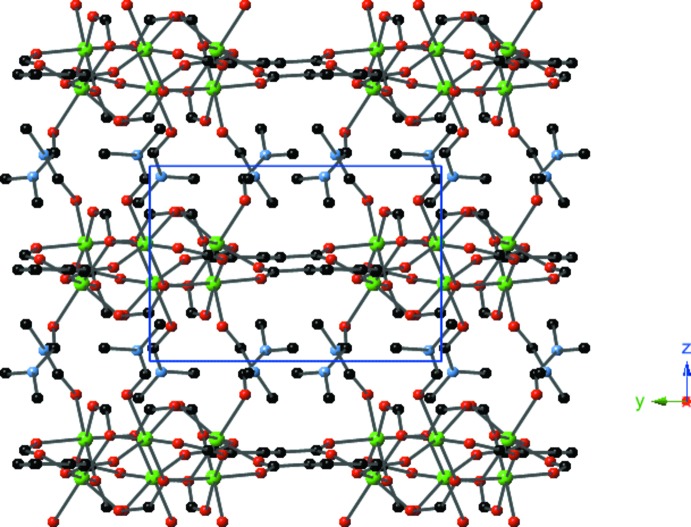
A portion of the packing diagram of (I) viewed along the a aixs.

## supplementary crystallographic information

### 1. Comment

Solvothermal reactions between Zn^II^ ion and simple organic ligands such as benzene-1,4-di­carb­oxy­lic acid (H_2_BDC) and benzene-1,3,5-tri­carb­oxy­lic acid (H_3_BTC) produce prototype metal-organic frameworks known as MOF-2, MOF-3, MOF-4, and MOF-5 (Eddaoudi *et al.*, 2000). Among them, MOF-4 formulated as [Zn_2_(BTC)(NO_3_)](C_2_H_5_OH)_5_(H_2_O) has dinuclear zinc clusters that are held by three carboxyl­ate groups from three distinct BTC ligands. In our trials to make a metal-organic framework composed of Zn^II^ paddle-wheel clusters and BTC, the title compound was obtained as single crystals. During a solvothermal reaction, decomposition of *N*,*N*-di­methyl­formamide produced formate which became a component of the title compound.

The title compound, (I) (Fig. 1), is isostructural to the related compounds with Ni^II^ (Maniam & Stock, 2011) and Mg^II^ (Yeh *et al.*, 2010). The crystal packing of (I) is shown in Figs. 2 & 3.

### 2. Experimental

The title compound was obtained by a solvothermal reaction between zinc(II) nitrate tetra­hydrate (0.157 g, 0.60 mmol) and benzene-1,3,5-tri­carb­oxy­lic acid (0.084 g, 0.40 mmol) in *N*,*N*-di­methyl­formamide (2.0 ml). When a sealed glass tube containing the reaction mixture was heated at 130 °C and for 24 h, the title compound was produced as colorless hexagonal columnar crystals.

### 3. Refinement

Hydrogen atoms were placed at calculated positions (C—H = 0.95–0.98 Å) and refined as riding, with U_iso_(H) = 1.2–1.5 U_eq_(C).

### Crystal data

**Table:**

[Zn_3_(HCO_2_)_3_(C_9_H_3_O_6_)(C_3_H_7_NO)_3_]	*D*_x_ = 1.888 Mg m^−^^3^
*M**_r_* = 757.56	Mo *K*α radiation, λ = 0.71073 Å
Trigonal, *P*3	Cell parameters from 2269 reflections
Hall symbol: -P 3	θ = 2.4–28.2°
*a* = 13.8594 (17) Å	µ = 2.76 mm^−^^1^
*c* = 8.0100 (14) Å	*T* = 153 K
*V* = 1332.5 (4) Å^3^	Column, colorless
*Z* = 2	0.10 × 0.02 × 0.02 mm
*F*(000) = 768	

### Data collection

**Table:**

Bruker SMART APEX CCD diffractometer	2027 independent reflections
Radiation source: fine-focus sealed tube	1563 reflections with *I* > 2σ(*I*)
Graphite monochromator	*R*_int_ = 0.153
phi and ω scans	θ_max_ = 27.5°, θ_min_ = 1.7°
Absorption correction: multi-scan (*SADABS*; Sheldrick, 2003)	*h* = −17→17
*T*_min_ = 0.770, *T*_max_ = 0.947	*k* = −11→18
7964 measured reflections	*l* = −9→10

### Refinement

**Table:**

Refinement on *F*^2^	0 restraints
Least-squares matrix: full	0 constraints
*R*[*F*^2^ > 2σ(*F*^2^)] = 0.040	H-atom parameters constrained
*wR*(*F*^2^) = 0.089	*w* = 1/[σ^2^(*F*_o_^2^) + (0.0317*P*)^2^ + 0.1686*P*] where *P* = (*F*_o_^2^ + 2*F*_c_^2^)/3
*S* = 0.81	(Δ/σ)_max_ < 0.001
2027 reflections	Δρ_max_ = 0.72 e Å^−^^3^
127 parameters	Δρ_min_ = −0.79 e Å^−^^3^

### Special details

**Table:**

Geometry. Geometry. All e.s.d.'s (except the e.s.d. in the dihedral angle between two l.s. planes) are estimated using the full covariance matrix. The cell e.s.d.'s are taken into account individually in the estimation of e.s.d.'s in distances, angles and torsion angles; correlations between e.s.d.'s in cell parameters are only used when they are defined by crystal symmetry. An approximate (isotropic) treatment of cell e.s.d.'s is used for estimating e.s.d.'s involving l.s. planes.Refinement. Refinement of F^2^ against ALL reflections. The weighted *R*-factor *wR* and goodness of fit S are based on F^2^, conventional *R*-factors *R* are based on F, with F set to zero for negative F^2^. The threshold expression of F^2^ > 2sigma(F^2^) is used only for calculating *R*-factors(gt) *etc*. and is not relevant to the choice of reflections for refinement. *R*-factors based on F^2^ are statistically about twice as large as those based on F, and R– factors based on ALL data will be even larger.

### Fractional atomic coordinates and isotropic or equivalent isotropic displacement parameters (Å^2^)

**Table:**

	*x*	*y*	*z*	*U*_iso_*/*U*_eq_	
Zn1	0.01272 (3)	0.23313 (3)	0.39911 (5)	0.01217 (12)	
O1	−0.25265 (17)	0.12229 (17)	0.4805 (3)	0.0160 (5)	
O2	−0.10070 (17)	0.28429 (17)	0.4230 (3)	0.0149 (5)	
O3	−0.05005 (17)	0.13366 (17)	0.6162 (3)	0.0140 (5)	
O4	0.10436 (18)	0.19215 (19)	0.7680 (3)	0.0162 (5)	
C1	−0.2019 (3)	0.2249 (2)	0.4582 (4)	0.0115 (6)	
C2	−0.2697 (3)	0.2816 (2)	0.4691 (4)	0.0113 (6)	
C3	−0.3856 (3)	0.2180 (3)	0.4700 (3)	0.0130 (7)	
H3	−0.4213	0.1390	0.4712	0.016*	
C4	0.0026 (3)	0.1477 (3)	0.7513 (4)	0.0140 (7)	
H4	−0.0408	0.1214	0.8502	0.017*	
O5	0.07684 (19)	0.32946 (18)	0.1759 (3)	0.0198 (5)	
N1	0.0391 (2)	0.3985 (2)	−0.0540 (3)	0.0204 (6)	
C5	0.0119 (3)	0.3253 (3)	0.0664 (4)	0.0203 (8)	
H5	−0.0626	0.2651	0.0699	0.024*	
C6	0.1529 (3)	0.4921 (3)	−0.0652 (5)	0.0286 (9)	
H6A	0.1890	0.5051	0.0442	0.043*	
H6B	0.1514	0.5590	−0.1003	0.043*	
H6C	0.1948	0.4749	−0.1470	0.043*	
C7	−0.0372 (3)	0.3837 (3)	−0.1897 (4)	0.0294 (9)	
H7A	−0.1092	0.3163	−0.1697	0.044*	
H7B	−0.0061	0.3762	−0.2956	0.044*	
H7C	−0.0477	0.4485	−0.1951	0.044*	

### Atomic displacement parameters (Å^2^)

**Table:**

	*U*^11^	*U*^22^	*U*^33^	*U*^12^	*U*^13^	*U*^23^
Zn1	0.0082 (2)	0.0087 (2)	0.0197 (2)	0.00431 (16)	0.00015 (15)	0.00033 (15)
O1	0.0128 (12)	0.0101 (11)	0.0253 (13)	0.0060 (10)	−0.0020 (10)	0.0021 (10)
O2	0.0088 (11)	0.0097 (11)	0.0276 (12)	0.0056 (9)	0.0017 (10)	−0.0001 (10)
O3	0.0093 (11)	0.0117 (11)	0.0179 (11)	0.0029 (9)	−0.0024 (9)	0.0013 (9)
O4	0.0109 (12)	0.0182 (12)	0.0194 (12)	0.0072 (10)	−0.0014 (10)	−0.0016 (10)
C1	0.0109 (16)	0.0078 (15)	0.0143 (16)	0.0036 (13)	−0.0041 (13)	−0.0021 (13)
C2	0.0106 (15)	0.0107 (15)	0.0129 (15)	0.0056 (13)	−0.0010 (13)	0.0011 (13)
C3	0.0135 (16)	0.0102 (16)	0.0144 (16)	0.0053 (13)	0.0001 (13)	0.0008 (13)
C4	0.0128 (16)	0.0087 (15)	0.0205 (17)	0.0053 (14)	0.0010 (14)	−0.0004 (13)
O5	0.0162 (12)	0.0180 (12)	0.0221 (13)	0.0063 (11)	0.0026 (10)	0.0074 (10)
N1	0.0216 (16)	0.0224 (16)	0.0208 (15)	0.0137 (14)	−0.0002 (13)	0.0020 (13)
C5	0.0178 (18)	0.0160 (18)	0.027 (2)	0.0084 (15)	0.0040 (16)	0.0004 (15)
C6	0.029 (2)	0.028 (2)	0.028 (2)	0.0127 (18)	0.0057 (17)	0.0075 (17)
C7	0.036 (2)	0.038 (2)	0.0217 (19)	0.025 (2)	−0.0024 (18)	−0.0004 (17)

### Geometric parameters (Å, º)

**Table:**

Zn1—O1^i^	2.028 (2)	C2—C3	1.394 (4)
Zn1—O2	2.031 (2)	C3—C2^iv^	1.380 (4)
Zn1—O4^ii^	2.105 (2)	C3—H3	0.9500
Zn1—O3^i^	2.108 (2)	C4—H4	0.9500
Zn1—O3	2.117 (2)	O5—C5	1.237 (4)
Zn1—O5	2.140 (2)	N1—C5	1.311 (4)
O1—C1	1.245 (3)	N1—C7	1.458 (4)
O1—Zn1^ii^	2.028 (2)	N1—C6	1.460 (5)
O2—C1	1.253 (4)	C5—H5	0.9500
O3—C4	1.265 (4)	C6—H6A	0.9800
O3—Zn1^ii^	2.108 (2)	C6—H6B	0.9800
O4—C4	1.232 (4)	C6—H6C	0.9800
O4—Zn1^i^	2.105 (2)	C7—H7A	0.9800
C1—C2	1.499 (4)	C7—H7B	0.9800
C2—C3^iii^	1.380 (4)	C7—H7C	0.9800
			
O1^i^—Zn1—O2	87.25 (9)	C3—C2—C1	119.7 (3)
O1^i^—Zn1—O4^ii^	168.88 (9)	C2^iv^—C3—C2	120.1 (3)
O2—Zn1—O4^ii^	93.23 (9)	C2^iv^—C3—H3	120.0
O1^i^—Zn1—O3^i^	90.61 (8)	C2—C3—H3	120.0
O2—Zn1—O3^i^	177.54 (9)	O4—C4—O3	126.7 (3)
O4^ii^—Zn1—O3^i^	89.09 (8)	O4—C4—H4	116.6
O1^i^—Zn1—O3	96.06 (9)	O3—C4—H4	116.6
O2—Zn1—O3	90.70 (8)	C5—O5—Zn1	119.8 (2)
O4^ii^—Zn1—O3	95.05 (8)	C5—N1—C7	122.1 (3)
O3^i^—Zn1—O3	88.32 (10)	C5—N1—C6	119.8 (3)
O1^i^—Zn1—O5	85.20 (9)	C7—N1—C6	117.7 (3)
O2—Zn1—O5	90.77 (9)	O5—C5—N1	124.4 (3)
O4^ii^—Zn1—O5	83.68 (8)	O5—C5—H5	117.8
O3^i^—Zn1—O5	90.26 (9)	N1—C5—H5	117.8
O3—Zn1—O5	178.11 (9)	N1—C6—H6A	109.5
C1—O1—Zn1^ii^	135.5 (2)	N1—C6—H6B	109.5
C1—O2—Zn1	126.83 (19)	H6A—C6—H6B	109.5
C4—O3—Zn1^ii^	120.2 (2)	N1—C6—H6C	109.5
C4—O3—Zn1	125.8 (2)	H6A—C6—H6C	109.5
Zn1^ii^—O3—Zn1	113.73 (10)	H6B—C6—H6C	109.5
C4—O4—Zn1^i^	132.5 (2)	N1—C7—H7A	109.5
O1—C1—O2	126.2 (3)	N1—C7—H7B	109.5
O1—C1—C2	116.5 (3)	H7A—C7—H7B	109.5
O2—C1—C2	117.3 (3)	N1—C7—H7C	109.5
C3^iii^—C2—C3	119.9 (3)	H7A—C7—H7C	109.5
C3^iii^—C2—C1	120.3 (3)	H7B—C7—H7C	109.5
			
Zn1^ii^—O1—C1—O2	42.6 (5)	C3^iii^—C2—C3—C2^iv^	1.0 (6)
Zn1^ii^—O1—C1—C2	−139.5 (2)	C1—C2—C3—C2^iv^	−175.8 (2)
Zn1—O2—C1—O1	−2.2 (5)	Zn1^i^—O4—C4—O3	−21.4 (5)
Zn1—O2—C1—C2	179.99 (19)	Zn1^ii^—O3—C4—O4	159.8 (3)
O1—C1—C2—C3^iii^	170.7 (3)	Zn1—O3—C4—O4	−26.7 (4)
O2—C1—C2—C3^iii^	−11.3 (4)	Zn1—O5—C5—N1	163.2 (2)
O1—C1—C2—C3	−12.5 (4)	C7—N1—C5—O5	173.5 (3)
O2—C1—C2—C3	165.5 (3)	C6—N1—C5—O5	0.5 (5)

Symmetry codes: (i) *y*, −*x*+*y*, −*z*+1; (ii) *x*−*y*, *x*, −*z*+1; (iii) −*y*, *x*−*y*+1, *z*; (iv) −*x*+*y*−1, −*x*, *z*.
